# Anaphylactic tests in model tumour antigen investigations.

**DOI:** 10.1038/bjc.1965.77

**Published:** 1965-09

**Authors:** M. M. Dale


### ANAPHYLACTIC TESTS IN MODEL TUMOUR ANTIGEN INVESTIGATIONS

M. MAUREEN DALE

*From the Department of Pharmacology, University College, London*

Received for publication February 24, 1965

ANAPHYLACTIC tests are among the many immunological techniques which have been used as analytical tools in the investigation of tumours. Makari (1955) suggested that anaphylactic phenomena could be used as the basis of a diagnostic test for cancer. He postulated that there was an antigen common to all human carcinomata and that this antigen would be present in the serum of patients with cancer. Guinea-pigs sensitized with human tumour extracts should therefore form antibodies not only to the species-specific antigens, the tissue antigens, and the serum antigens in the extract, but also to the tumour-specific antigens. The isolated uteri of such guinea-pigs should give Dale-Schultz reactions when challenged with normal serum, and then when fully desensitized to normal serum should give a further positive response with cancer serum. Makari did not do any preliminary studies on the limits of sensitivity and accuracy of the Dale–Schultz test used in this context, but applied the test, *ab initio*, in the clinical field. He claimed that it gave 96·8 per cent positives in known cancer cases. Burrows (1958) reported similar results. Hackett and Gardonyi (1960), using a more carefully considered technique reported only 47 per cent positive results with cancer sera and no false positives with normal sera. Less conclusive results were reported by McEwen (1959), Maass and Schniewind (1960) and Wittig, Teichmann and Schneeweiss (1962).

The technique is a complicated one with many variables and it is not easy to assess these conflicting reports in the literature. Negative results may mean either that there are no tumour-specific antigens of the sort postulated or that the tests used were not capable of detecting such antigens, or both. It is not practicable to use anaphylactic tests to investigate the wider problem of the existence of tumour-specific antigens until more detailed information is available on the limits of efficacy of such tests.

In previous studies (Dale, 1965*a*, *b*) some of the assumptions on which the use of anaphylactic tests were based were investigated and found to be invalid. Certain general operational limits were outlined within which the tests might possibly be used to analyse antigen mixtures. With these limits in mind a study has been carried out to assess whether if a cancer antigen were present in tissues and serum, it could in fact be detected by anaphylactic techniques. A simple model of Makari's diagnostic test was designed, using rat tissue and rat serum. A known antigen, bovine $\gamma$-globulin, was selected to represent the postulated tumour antigen. Mock tumour extracts were made up consisting of bovine $\gamma$-globulin (B$\gamma$G) mixed in various proportions with an extract of normal rat liver. Guinea-pigs were sensitized with this mixture and after a suitable interval the anaphylactic responses of isolated organs were investigated. The tests used were the Dale

Schultz reaction, which had been used by many investigators, and the measurement of histamine released from samples of chopped lung, which had not been applied to this problem before. In the actual tests, pooled normal rat serum was used for desensitization and then the response to mock cancer serum (normal rat serum + BγG), was determined.

### MATERIALS AND METHODS

Two groups of guinea-pigs were sensitized with different concentrations of BγG in rat liver extract. A 25 % extract was prepared by grinding up a weighed quantity of fresh rat liver with sand, with a pestle and mortar, adding the requisite quantity of distilled water and leaving the mixture to stand for 2 hours. NaCl was then added to make the solution isotonic and the extract was centrifuged at 3000 r.p.m. for 30 minutes. Kjeldahl estimations of total N content of the extracts were done and (assuming the N content in protein to be 16 %) the protein concentration was calculated. The following antigen mixtures were used for sensitization :

Group I (7 guinea-pigs) : 2·5 mg. rat liver protein + 50 μg. BγG

Group II (5 guinea-pigs) : 500 μg. rat liver protein + 100 μg. BγG

The antigen mixture was emulsified with an equal quantity of incomplete or complete Freund's adjuvants. In sensitizing the guinea-pigs the emulsion was injected intradermally into 2 sites, behind each ear, a total volume of 0·5 ml. per animal being given.

The details of the performance of the Dale–Schultz tests and the measurement of histamine-release from chopped lung have been given in a previous paper (Dale, 1965*a*).

Three " mock cancer " test sera, A, B and C, were made up for use in the tests. These consisted of three different concentrations of BγG, 1 %, 0·1 % and 0·01 %, in pooled dialysed normal rat serum. The normal rat serum contained 50 mg./ml. serum protein, and the amount of " mock cancer antigen " was therefore equivalent to $\frac{1}{5}$, $\frac{1}{50}$ and $\frac{1}{500}$ of the normal serum proteins in test sera A, B and C respectively. These sera were then diluted for the anaphylactic tests, as given below. Both tests were done on all 12 animals.

*The Dale–Schultz tests.*—The loops of ileum were desensitized to normal serum 0·1 % (final concentration) and then challenged with 0·1 % of one of the sera containing BγG. With these dilutions the BγG concentrations were $10^{-5}$ in A, $10^{-6}$ in B and $10^{-7}$ in C. As in previous experiments a response to the second antigen, the " test serum ", was only rated as positive if it were more than 10 % of the maximum response. With each guinea-pig the response to BγG alone in the relevant concentrations, $10^{-7}$, $10^{-6}$ and $10^{-5}$, was tested in separate loops.

*The histamine-release tests.*—The basic technique of measurement of histamine-release from chopped lung was described by Mongar and Schild (1953, 1957, 1960). Its general application for discrimination between antigens was discussed in a previous paper (Dale, 1965*b*). In the present study the following protocol was used :

| | Sample numbers | First challenge (partial desensitization) | Second challenge |
|---|---|---|---|
| Control samples . | 1, 2, 3, 4 | 0·1% normal serum | 0·1% normal serum |
| Test samples . | 5, 6, 7, 8 | 0·1% normal serum | 0·1% " test serum" A or B |
| Test samples . | 9, 10, 11, 12 | 0·1% normal serum | 0·1% " test serum " B or C |

All the samples were partially desensitized with 0·1% (final concentration) normal serum. Then, after washing, the amount of histamine released in the two sets of test samples 5, 6, 7, 8 and 9, 10, 11, 12 by the test sera was compared with the amount released on second challenge with normal serum in control samples 1, 2, 3, 4 on a *t*-test. A result was rated as positive if there were a significant difference between the control and the test samples at the level $P = 0{\cdot}025$. In addition, histamine release with BγG alone, in the relevant concentrations, was measured.

The solutions used were tested on normal tissues and found to cause no histamine release and no Dale–Schultz reaction. The effects of BγG on the tissues of a guinea-pig sensitized to liver extract alone, and the effect of normal serum on the tissues of a guinea-pig sensitized to BγG alone, were tested and found to be insignificant.

### RESULTS

Both tests were done on each guinea-pig. All the animals proved to be strongly sensitized to the main antigens in the sensitizing mixture as evidenced by marked reactions with rat serum in both tests. There were then two main questions to be answered :

(a) Had the animals become sensitized to BγG as well?

(b) In those animals which showed evidence of sensitivity to BγG, was there a positive anaphylactic response to the test serum after desensitization with normal serum, i.e. was the BγG in the test serum detected by the anaphylactic tests?

### *Dale–Schultz tests*

The results are given in Table I and show the following :

(a) *Sensitivity to BγG alone.*—In group I, where the BγG had formed approximately $\frac{1}{50}$ of the sensitizing mixture, only 3 out of 7 animals showed sensitivity to

TABLE I.—*The Detection of Mock Cancer Antigen (BγG) in Rat Serum with the Dale–Schultz Test*

The Dale-Schultz response is given as percentage of the maximum response

| Group | Ratio of sensitizing antigens | Guinea-pig No. | Evidence of sensitivity to " cancer antigen " alone: A (BγG $10^{-5}$) | B (BγG $10^{-6}$) | C (BγG $10^{-7}$) | Dale-Schultz response (after prior desensitization to normal serum 0·1%) with: Test serum A (0·1%) | Test serum B (0·1%) | Test serum C (0·1%) |
|---|---|---|---|---|---|---|---|---|
| I | BγG 50 μg. | 1 | — | — | .. | 0 (−) | 0 (−) | .. |
| | Liver : 2·5 mg. | 2 | √ | √ | .. | 0 (−) | 0 (−) | .. |
| | protein | 3 | √ | √ | .. | 0 (−) | 0 (−) | .. |
| | | 4 | — | — | .. | 0 (−) | 0 (−) | .. |
| | | 5 | √ | √ | .. | 96 (+) | 65 (+) | .. |
| | | 6 | — | — | .. | 0 (−) | 0 (−) | .. |
| | | 7 | — | — | .. | 0 (−) | 0 (−) | .. |
| II | BγG 100 μg. | 8 | √ | √ | .. | 100 (+) | 92 (+) | .. |
| | Liver : 500 μg. | 9 | .. | √ | √ | 65 (+) | 58 (+) | 15 (+) |
| | protein | 10 | .. | √ | √ | .. | 72 (+) | 20 (+) |
| | | 11 | .. | √ | √ | .. | 41 (+) | 7 (−) |
| | | 12 | .. | √ | √ | .. | 30 (+) | 30 (+) |

TABLE II.—*The Detection of Mock Cancer Antigen (BγG) in Rat Serum by the Histamine-Release Test*

Histamine release is given as percentage of total histamine* per sample. Each figure is the mean of 4 samples. The guinea-pigs were the same as those listed in Table I.

| Group | Ratio of sensitizing antigens | Guinea-pig No. | Evidence of sensitivity to "cancer antigen" alone: A (BγG $10^{-5}$) | B (BγG $10^{-6}$) | C (BγG $10^{-7}$) | Histamine release (after prior desensitization to normal serum 0·1%) with: Normal serum (0·1%) | Test serum A (0·1%) | Test serum B (0·1%) | Test serum C (0·1%) |
|---|---|---|---|---|---|---|---|---|---|
| I | BγG : Liver protein 50 μg. : 2·5 mg. | 1 | √ | — | .. | 3·3 | 3·4 (−) | 3 (−) | .. |
| | | 2 | √ | — | .. | 2·9 | 3·4 (−) | 3·1 (−) | .. |
| | | 3 | √ | √ | .. | 3·8 | 4·9 (+) | 5·3 (+) | .. |
| | | 4 | — | — | .. | 4·0 | 4 (−) | 4·2 (−) | .. |
| | | 5 | √ | √ | .. | 3·1 | 6·2 (+) | 4·6 (+) | .. |
| | | 6 | √ | √ | .. | 5·0 | 5·5 (−) | 5·1 (−) | .. |
| | | 7 | √ | — | .. | 1·1 | 0·8 (−) | 0·6 (−) | .. |
| II | BγG : Liver protein 100 μg. : 500 μg. | 8 | √ | √ | .. | 1·4 | 13·3 (+) | 10·2 (+) | .. |
| | | 9 | .. | √ | √ | 2·5 | — | 4·8 (+) | 7·7 (+) |
| | | 10 | .. | √ | √ | 3·1 | .. | 4·1 (+) | 3·5 (−) |
| | | 11 | .. | √ | √ | 1·2 | .. | 6·2 (+) | 2·8 (+) |
| | | 12 | .. | √ | √ | 1·8 | .. | 3·6 (+) | 2·5 (+) |

* The "total histamine" refers to the histamine released by antigen + the residual histamine (released by subsequent boiling of the tissue).

BγG. In group II, where BγG had formed $\frac{1}{6}$ of the sensitizing mixture all 5 animals tested showed sensitivity to BγG.

(b) *Response to the test sera after desensitization to normal serum.*—In group I, of the 3 animals in this group which had shown sensitivity to BγG when given alone, only one animal gave a positive Dale–Schultz reaction with serum containing BγG after desensitization with normal serum. In group II, all 5 animals used showed sensitivity to BγG given alone, and after desensitization with normal serum, 2 out of 2 animals tested with " test serum A ", 5 out of 5 animals tested with " test serum B ", and 3 out of 4 animals tested with " test serum C " gave positive Dale–Schultz reactions.

Thus in group I, 12 out of 14 tests gave " false negative " results and in group II, 1 out of 11 tests gave a " false negative " result.

### *Histamine-release tests*

The results are given in Table II and show the following :

(a) *Sensitivity to BγG alone.*—In group I, 6 out of 7 animals, and in group II, 5 out of 5 animals had become sensitized to BγG.

(b) *Response to the test sera after desensitization to normal serum.*—In the smooth muscle experiments, for each test the whole procedure has usually to be carried out on one piece of ileum, because of the great variability between different loops. When chopped lung is used, on the other hand, numerous strictly comparable samples of sensitized tissue are available from each animal. The variation between these samples is small and is moreover measurable, and its effects on the results can be calculated. It is possible to use a larger number of samples for each test and, having partially desensitized all of them with normal serum, to administer cancer serum to half the samples (the test samples) and normal serum to the rest

(the control samples) and compare the difference in histamine release between the two sets of samples. The results obtained are amenable to statistical analysis, and within limits, the sensitivity of the test can be increased by increasing the number of samples. Using this procedure it was found that after desensitization to normal serum in group I, of the 6 animals which had become sensitized to the B$\gamma$G, 4 showed no difference in histamine-release between the test samples and the control samples, i.e. the test did not detect the B$\gamma$G. In the remaining two animals (No. 3 and 5) there was a positive response with test serum, i.e. the percentage histamine-release in the test samples was in each case significantly greater than in the control samples on a *t*-test at the 2·5 % level. In group II, after desensitization to normal serum, 5 out of 5 animals gave a positive response to test serum B and 3 out of 4 gave a positive response to test serum C.

The measurements of the amount of histamine released in each sample were done on the guinea-pig ileum preparation which is itself subject to variation, and it is possible that this could affect the final results. It is feasible to examine this possibility by doing an analysis of variance to determine whether the variation between the readings on an individual lung sample is significantly different from the variation between the results of different samples and/or the variation between the results of different treatments (i.e. exposure to normal or cancer serum). Analyses of variance were, in fact, done on the data from each experiment which had given a positive result. The experimental design consisted of nested samples and the model of the analysis of variance was from Snedecor (1956). In each case the variance ratios for

$$\frac{\text{between-treatment variation}}{\text{between-sample variation}} \quad \text{and} \quad \frac{\text{between-treatment variation}}{\text{between-reading variation}}$$

were significant at the 5 % level. It was decided therefore that the differences between treatments could be considered as real differences and not merely reflections of the variation between samples or the variation between readings.

### DISCUSSION

The present study sought to answer the question: " If a tumour antigen exists in a tumour and is also present in the serum—can it be detected by anaphylactic tests? " A $\gamma$-globulin was the substance chosen to represent the mock cancer antigen because several investigators have reported the presence of additional globulins (not necessarily tumour-specific) in the serum of tumour-bearing animals (Darcy, 1957; Miller and Bernfeld, 1960; Fine, Boffa and Zajdela, 1962; Abelev *et al.*, 1963). It was recognized that a preparation of $\gamma$-globulin would be likely to consist of more than one physico-chemical entity, but it was thought that for practical purposes the preparation could be treated as a single antigen. Porter (1960) has made the point that the antigenic specificity of $\gamma$-globulin in contrast to its physico-chemical properties shows a remarkable homogenicity in any one species.

Considering the results as a whole, the answer to the question posed appears to be that the antigen may very well not be detected by an anaphylactic test. When the mock tumour antigen, though given in adequate sensitizing dosage, formed a low proportion of the sensitizing mixture, it was very infrequently detected even when present in a high concentration of the challenging serum. It was only consistently detected when it had been given in high absolute dosage in, and had formed a relatively high proportion of, the sensitizing mixture, and was subsequently present in a concentration of at least $10^{-3}$ in the challenging serum. This does not accord with the claim made by Makari (1955) that these anaphylactic tests are highly sensitive in this context. It might be argued that a qualitative change in the serum of this magnitude could be detected by less complicated procedures.

The anaphylactic tests gave a high percentage of false negative results (i.e. results which were negative when the antigen *was* present). There appear to be two main reasons for false negative results :

(a) There may be inadequate sensitization to the " tumour antigen " in some animals. Many workers appear to have assumed that there would necessarily be good sensitization to all antigens in an antigen mixture. In a previous study, however (Dale, 1965*b*), it was pointed out that the presence of even one extra antigen in the sensitizing mixture may decrease the sensitivity of an anaphylactic test in detecting a particular antigen. It is apparent that this phenomenon occurred in these experiments. In several animals in group I, for example, there had been adequate sensitization to rat serum proteins but there had been no sensitization to BγG although the amount of BγG given in the sensitizing mixture was well within the dose range accepted as being reasonable for sensitization even without adjuvants (Kabat and Mayer, 1961) and well above that shown previously to give good sensitization when given alone (Dale, 1965*a*, *b*). The Dale–Schultz test gave poorer results than the histamine-release test in this respect: in group I, of the 7 animals sensitized to both BγG and rat liver extract, 4 showed no sensitivity to BγG with the Dale–Schultz test while only 1 showed no sensitivity to BγG with the histamine-release test.

(b) The tissues may be reasonably well sensitized to the mock tumour antigen, but the process of desensitization may exhaust the " anaphylactic potential " of the tissue. This phenomenon was pointed out in a previous study with a model system of mixed antigens (Dale, 1965*b*). In the present study a number of animals in group I, for example, showed evidence of good sensitization to the mock cancer antigen, BγG, as shown by the responses of preparations in which BγG was administered alone. But when, in other preparations from the same animals, the tissues were first desensitized with normal serum, there was no response subsequently to serum containing BγG even when the concentration of BγG was 10 mg./ml. of serum or equivalent to $\frac{1}{5}$ of the normal serum proteins.

In as far as one can extrapolate from a simple model study of this sort to experiments with real tumours, it would seem that it might conceivably be possible to use anaphylactic tests in this sort of tumour antigen study, but the chances of getting false negative results would be high unless the tumour antigen formed a very substantial proportion of the antigenic material in the tumour extract. It is probably unrealistic to expect that a tumour antigen, if present, would comprise as much as 15 % of the antigenic material in a tumour extract. Equally it is probably unrealistic to expect that in a tumour-bearing animal, a tumour antigen would be present in the serum in concentrations of the order of $10^{-3}$–$10^{-2}$. It would seem therefore that unless such a tumour antigen were very powerfully antigenic, anaphylactic tests would not be very useful tools in tumour antigen studies or in diagnostic tests. If the tests were to be used at all in this type of analysis of complex antigen mixtures, the histamine-release test, though more laborious to do, would be preferable to the Dale-Schultz test because the data obtained are amenable to statistical analysis and, within limits, the error of the technique can be calculated and taken into account.

### SUMMARY

The efficacy of anaphylactic tests in detecting " tumour " antigen in serum was investigated. A simple model of a tumour-antigen study was carried out using rat tissue and rat serum, with bovine $\gamma$-globulin (B$\gamma$G) acting as a mock cancer antigen.

It was found that if B$\gamma$G (absolute dosage 100 $\mu$g.) had formed $\frac{1}{6}$ of the antigen mixture used for sensitization it was readily detected when present in a concentration of $10^{-3}$ in the serum used for challenge, but not invariably detected in a concentration of $10^{-4}$. If B$\gamma$G (absolute dosage 50 $\mu$g.) had formed approximately $\frac{1}{50}$ of the sensitizing mixture, it was infrequently detected even when present in the challenging serum in a concentration of $10^{-2}$. It is concluded that anaphylactic tests used in this context do not have a very high sensitivity or discriminatory capacity.

I am indebted to Professor H. O. Schild and Dr. J. L. Mongar for valuable advice. This work was supported by a grant from the British Empire Cancer Campaign for Research.

### REFERENCES

ABELEV, G. I., PEROVA, S. D., KHRAMKOVA, N. I., POSTNIKOVA, Z. A. AND IRLIN, I. S.—(1963) *Transplantation*, **1**, 174.
BURROWS, D.—(1958) *Brit. med. J.*, i, 368.
DALE, M. M.—(1965*a*) *Immunology* (in press).—(1965*b*) *Ibid.* (in press).
DARCY, D. A.—(1957) *Brit. J. Cancer*, **11**, 137.
FINE, J. M., BOFFA, G. A., AND ZAJDELA, F.—(1962) *C.R. Acad. Sci., Paris*, **255**, 1045.
HACKETT, E. AND GARDONYI, E.—(1960) *Brit. med. J.*, i, 1785.
KABAT, E. A. AND MAYER, M. M.—(1961) ' Experimental Immunochemistry '. 2nd edition, Springfield, Illinois, U.S.A. (Charles C. Thomas) p. 269.
MAASS, H. AND SCHNIEWIND, H.—(1960) *Klin. Wschr.*, **38**, 1164.
MCEWEN, L. M.—(1959) *Brit. med. J.*, ii, 615.
MAKARI, J. G.—(1955) *Ibid.*, ii, 1291.
MILLER, E. E. AND BERNFELD, P.—(1960) *Cancer Res.*, **20**, 1149.
MONGAR, J. L. AND SCHILD, H. O.—(1953) *Brit. J. Pharmac. Chemother.*, **8**, 103.—(1957) *J. Physiol.*, **135**, 301.—(1960) *Ibid.*, **150**, 546.
PORTER, R. R.—(1960) ' The Plasma Proteins ', 1 ; edited by Putnam, F. W., New York (Academic Press, Inc.), p. 241.
SNEDECOR, G. W.—(1956) ' Statistical Methods ', 5th edition. Ames (Iowa State College Press). Section 10 : 14.
WITTIG, G., TEICHMANN, B. AND SCHNEEWEISS, U.—(1962) *Acta biol. med. germ.*, **8**, 274.


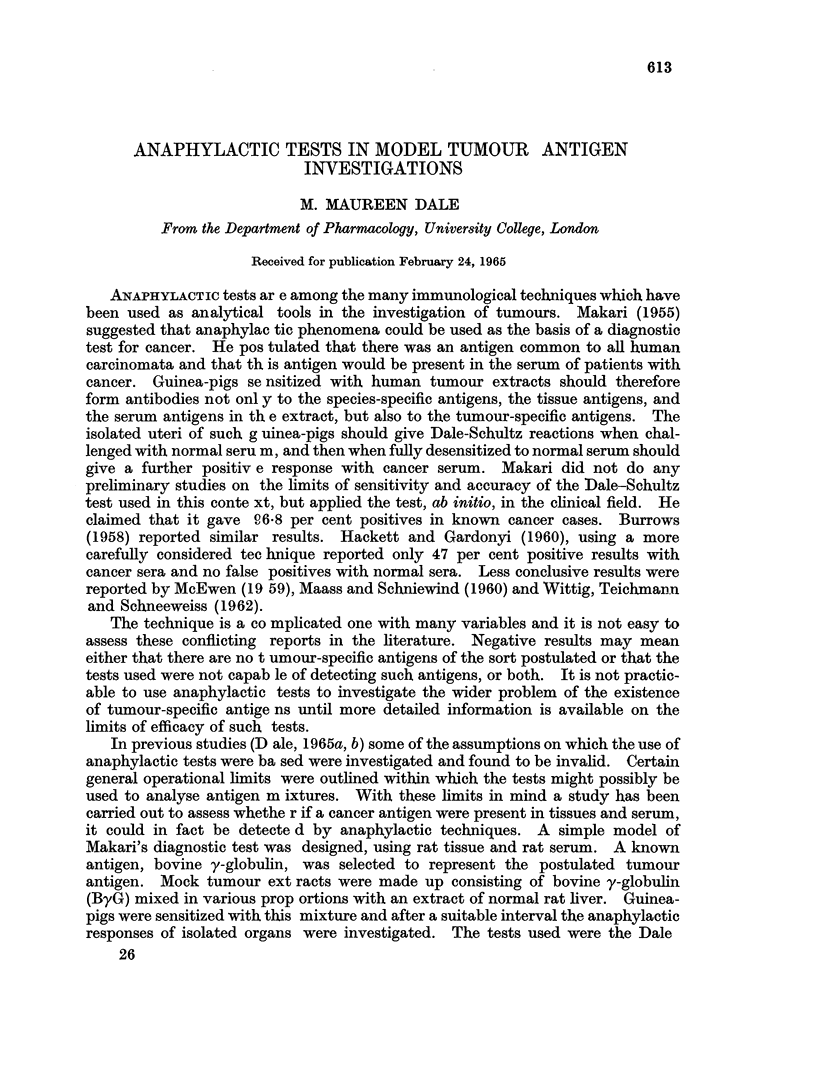

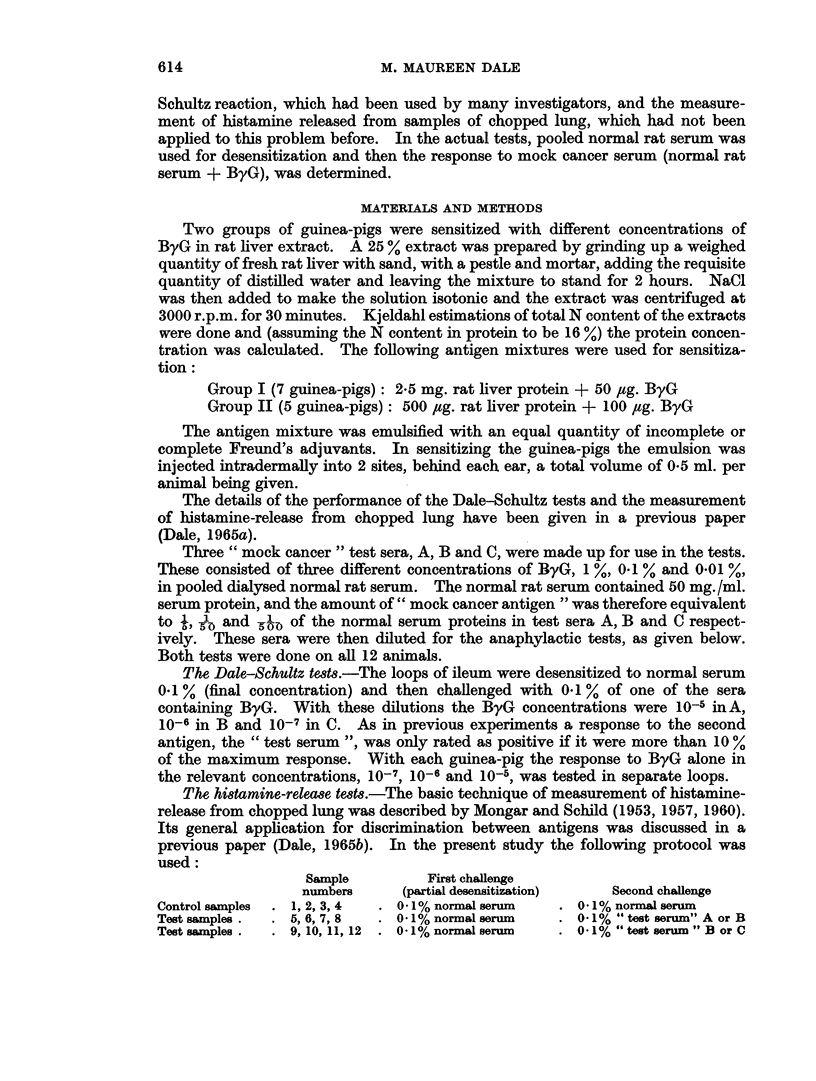

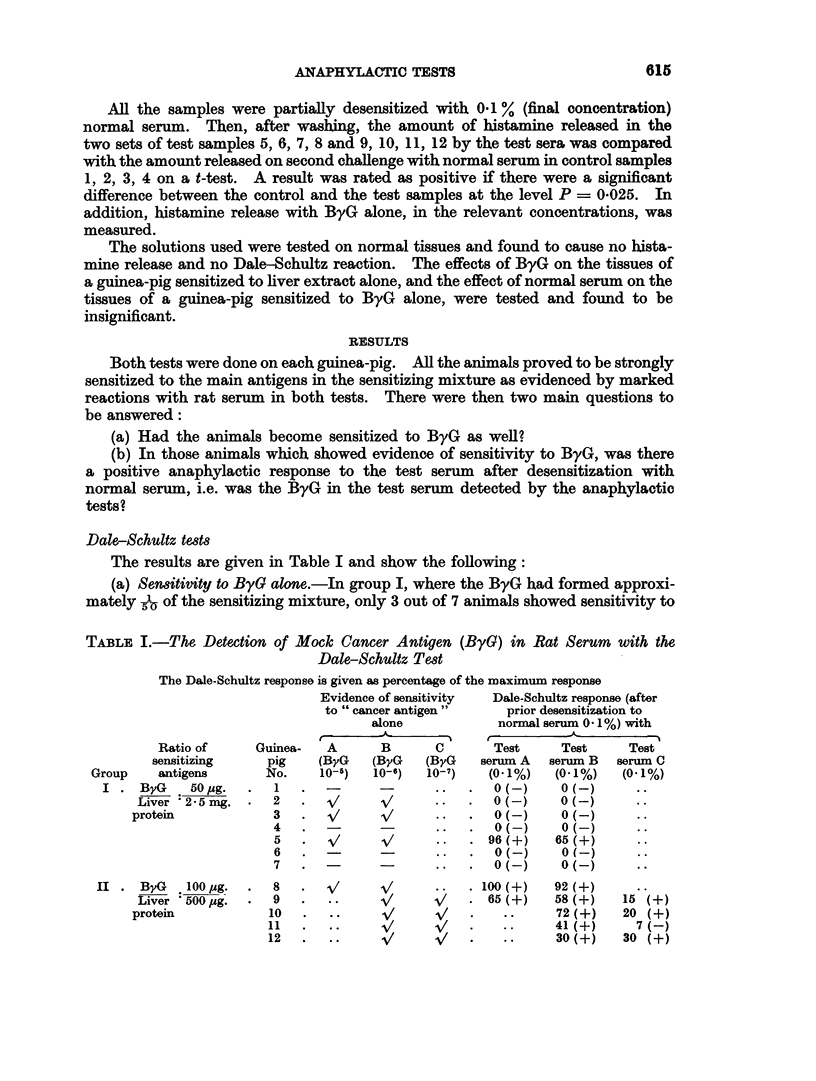

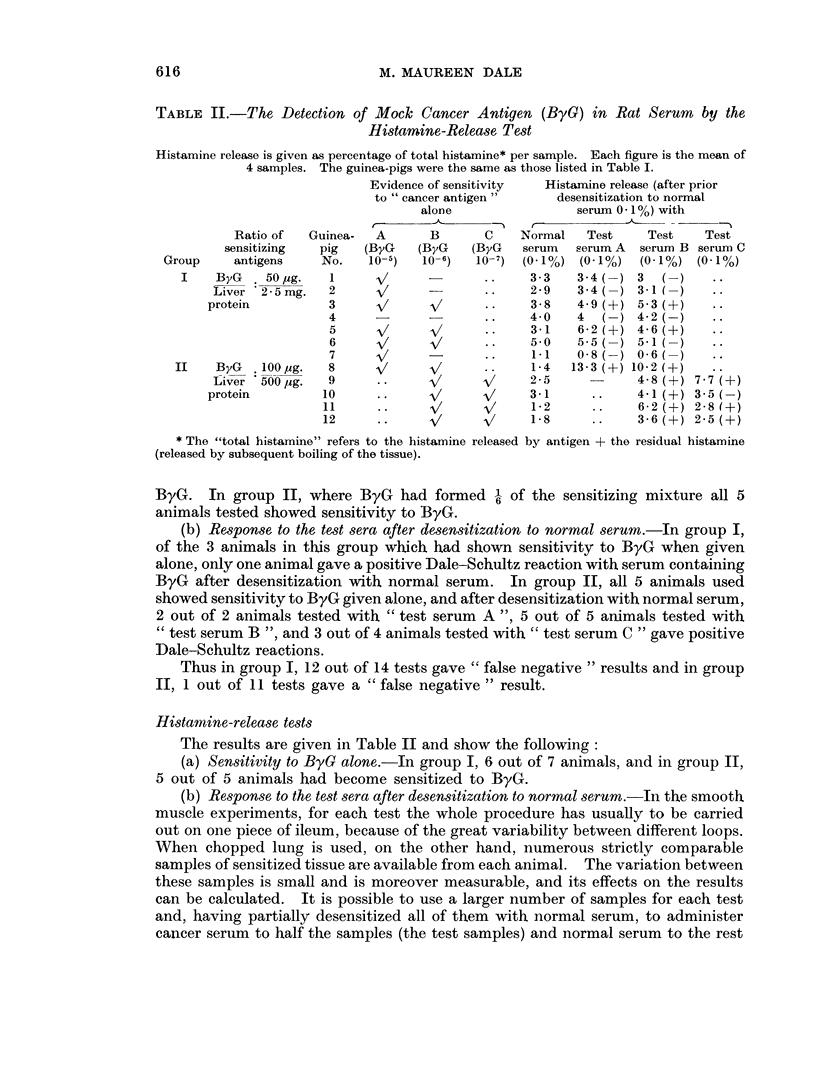

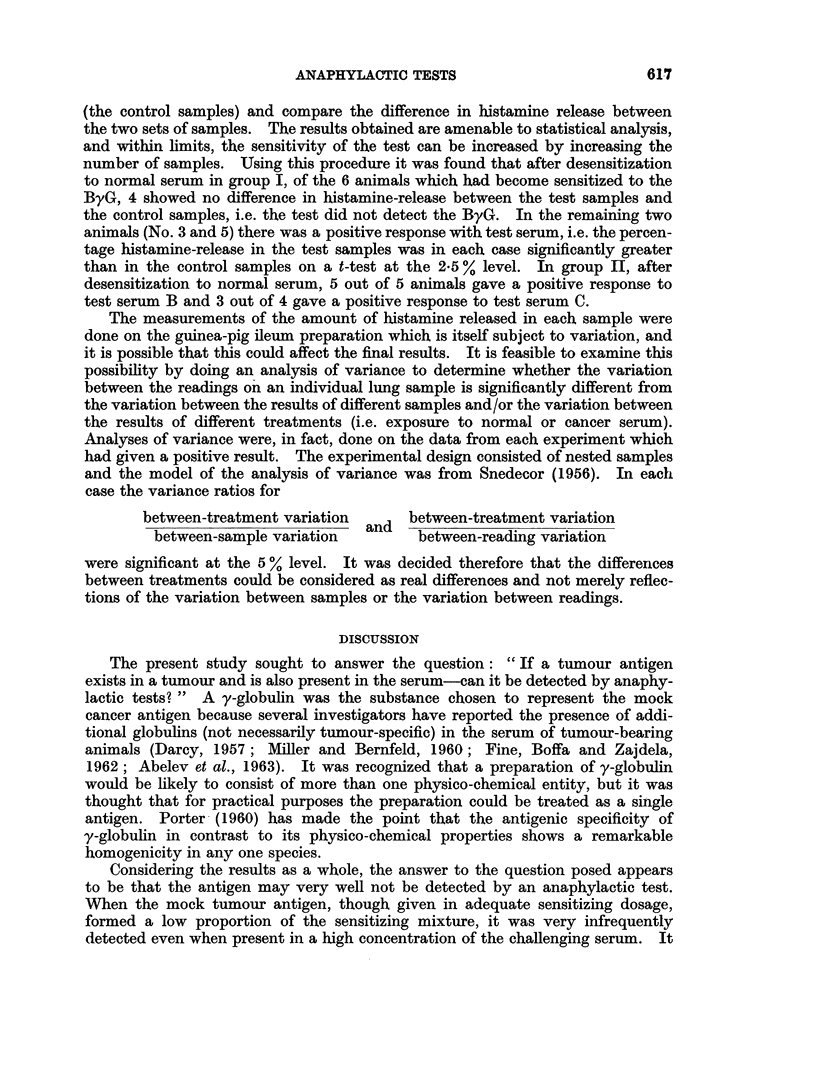

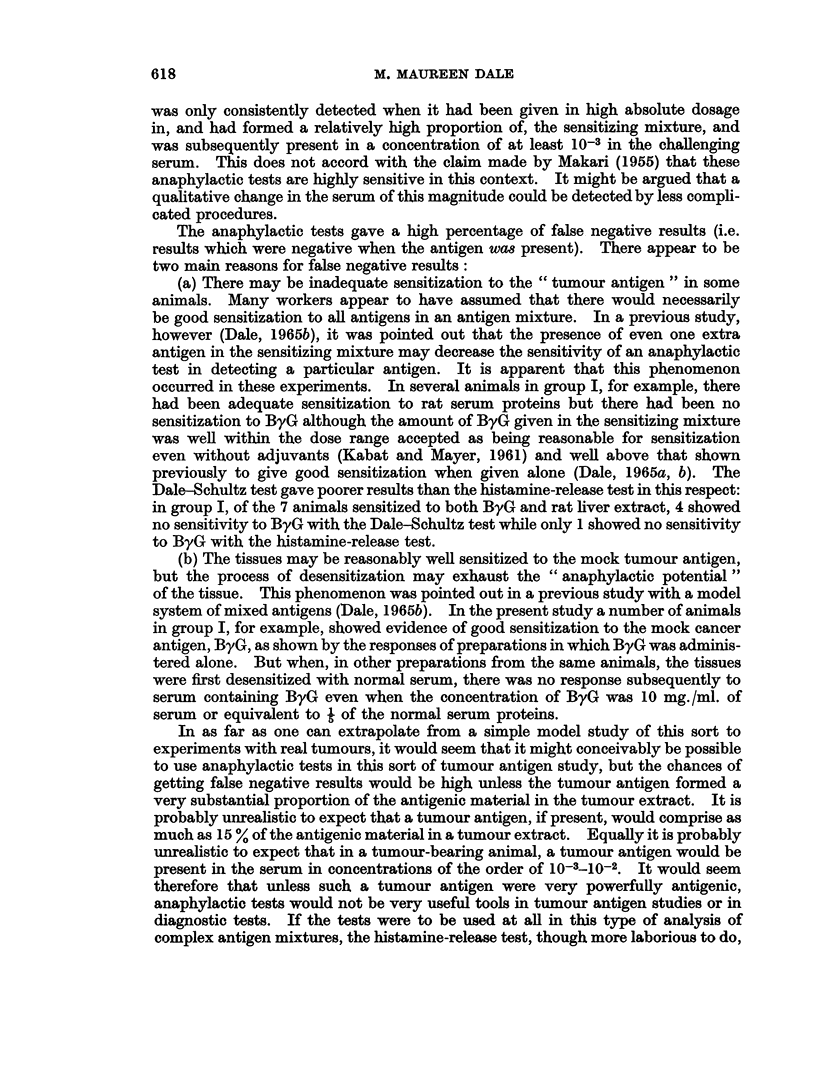

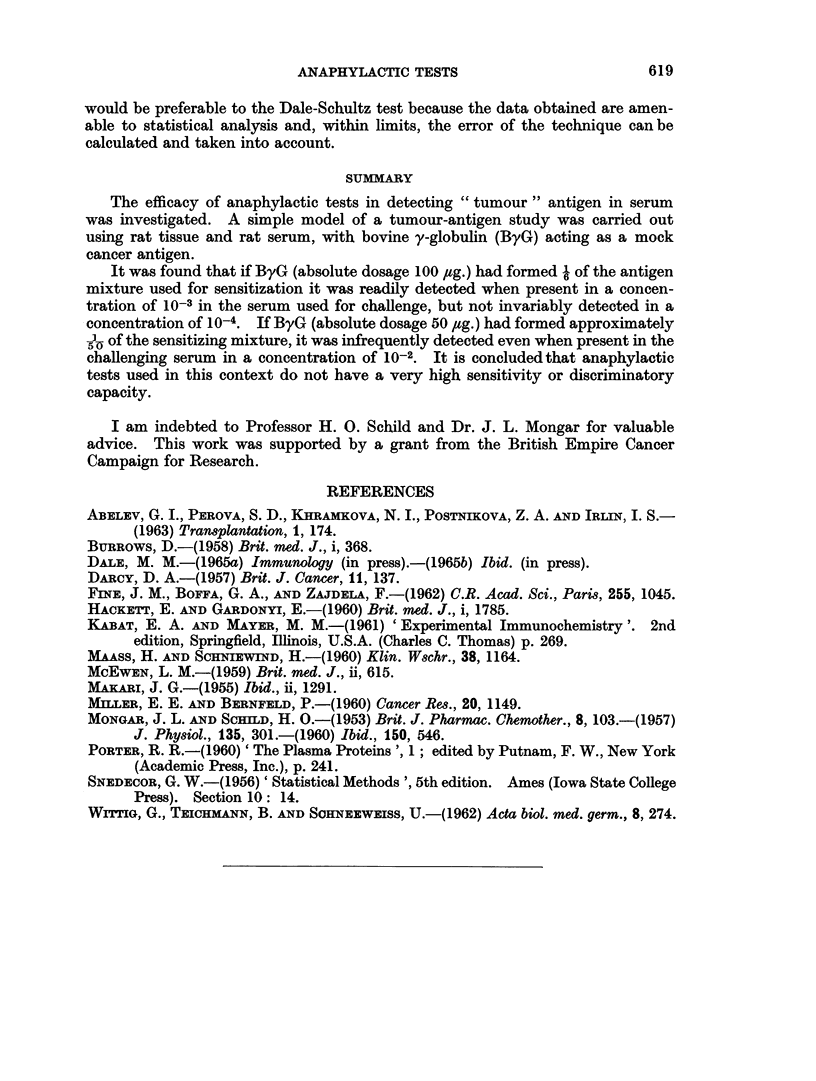


## References

[OCR_00412] ABELEV G. I., PEROVA S. D., KHRAMKOVA N. I., POSTNIKOVA Z. A., IRLIN I. S. (1963). Production of embryonal alpha-globulin by transplantable mouse hepatomas.. Transplantation.

[OCR_00416] BURROWS D. (1958). Schultz-Dale test for detection of specific antigen in sera of patients with carcinoma.. Br Med J.

[OCR_00417] DARCY D. A. (1957). Immunological demonstration of a substance in rat blood associated with tissue growth.. Br J Cancer.

[OCR_00422] HACKETT E., GARDONYI E. (1960). Serum detection of carcinoma; experience with the Schultz-Dale technique.. Br Med J.

[OCR_00429] MILLER E. E., BERNFELD P. (1960). Abnormal plasma components in C3H mice bearing spontaneous tumors.. Cancer Res.

[OCR_00431] MONGAR J. L., SCHILD H. O. (1960). A study of the mechanism of passive sensitization.. J Physiol.

[OCR_00443] WITTIG G., TEICHMANN B., SCHNEEWEISS U. (1962). [The suitability of the Schultz-Dale reaction for the demonstration of specific tumor antigens. A critical comment].. Acta Biol Med Ger.

